# Cytokine and reactivity profiles in SLE patients following anti-CD19 CART therapy

**DOI:** 10.1016/j.omtm.2023.08.023

**Published:** 2023-09-01

**Authors:** Daniel Nunez, Darshil Patel, Jenell Volkov, Steven Wong, Zachary Vorndran, Fabian Müller, Michael Aigner, Simon Völkl, Andreas Mackensen, Georg Schett, Samik Basu

**Affiliations:** 1 Department of Computational Biology, Cabaletta Bio, Philadelphia, PA, USA; 2 Department of Protein and Molecular Biology, Cabaletta Bio, Philadelphia, PA, USA; 3 Department of Translational Medicine, Cabaletta Bio, Philadelphia, PA, USA; 4 Department of Medicine 5, Haematology and Oncology, Friedrich Alexander University Erlangen-Nürnberg (FAU) and University Hospital Erlangen, Erlangen, Germany; 5 Department of Medicine 3, Rheumatology and Immunology, Friedrich Alexander University Erlangen-Nürnberg (FAU) and University Hospital Erlangen, Erlangen, Germany

**Keywords:** chimeric antigen receptor T cell, CAR, systemic lupus erythematous, SLE, cytokines, antibodies, vaccines, autoimmune disease

## Abstract

Chimeric antigen receptor (CAR) T cells targeting CD19^+^ B cells have demonstrated efficacy in refractory systemic lupus erythematosus (SLE). Although initial clinical data suggest that anti-CD19 CAR T cell therapy is well tolerated and highly effective, the immunologic consequences of CAR T cell therapy in SLE patients remain unclear. We profiled serum in six refractory SLE patients prior to and 3 months following CAR T cell infusion. Three months post T cell infusion, the inflammatory cytokines IL-6 and TNFα decreased in patient sera. This was accompanied by elevations in serum IL-7 and BAFF. Furthermore, SLE-associated antibodies dropped profoundly in five of six patients. Last, consistent with other reports of CD19 CAR T therapy in B cell malignancies, we were able to show marginal impact of anti-CD19 CART therapy on pre-existing humoral immune responses in SLE patients. Together, these results provide insights into the mechanisms of efficacy of anti-CD19 CAR T cell therapy in SLE.

## Graphical abstract

**Figure:**
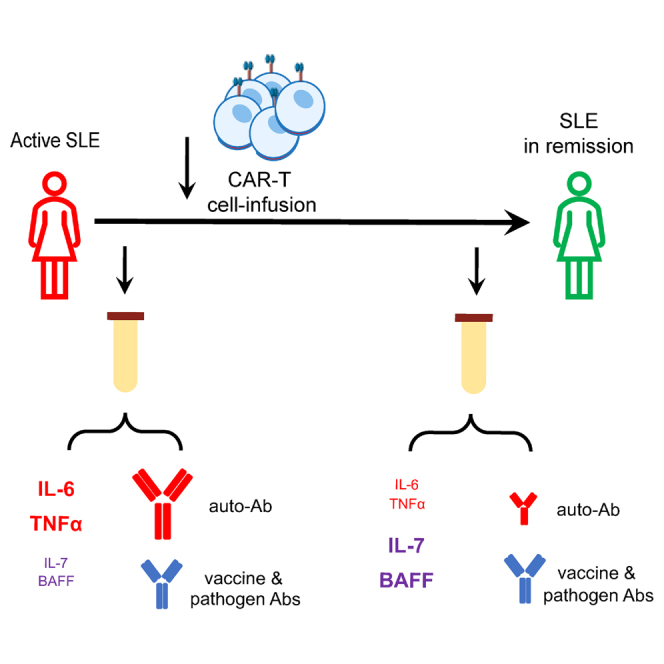

## Teaser

CD19 CART therapy in SLE patients results in multiple serological changes. First, there is a decrease in the inflammatory cytokines IL-6 and TNFα. This is accompanied by elevations in serum IL-7 and BAFF. Furthermore, SLE-associated antibodies decrease in most patients. Last, there is a marginal impact on pre-existing humoral immunity.

## Introduction

Systemic lupus erythematosus (SLE) is a severe, life-threatening autoimmune disease characterized by widespread immune activation, autoantibodies to self-antigens, and organ dysfunction.^1^ A breakdown in tolerance is believed to be the result of genetic and environmental factors culminating in the generation of autoreactive T and B lymphocytes.^1^ Autoreactive B cells play a dual role in SLE pathogenesis. In addition to producing autoantibodies that directly cause tissue damage, B cells also process and present self-antigen to autoreactive T cells that further mediate disease.^2^^,^^3^ As such, therapies that either antagonize or deplete B cells have been investigated in SLE.^4^^,^^5^ Although monoclonal antibodies targeting CD19 and CD20 have demonstrated benefit in subsets of patients, relapse is common.^4^^,^^5^ Resistance to monoclonal antibody therapy may be due to incomplete depletion of B cells residing within secondary lymphoid organs.^6^ Chimeric antigen receptor (CAR) T cells targeting CD19^+^ B cells have been shown to eliminate B cells both in the peripheral blood and within secondary lymphoid organs.^7^ Recently, anti-CD19 CAR T cell therapy has been used to treat immunosuppressive refractory SLE patients in the context of a point-of-care program.^8^ Initial clinical and correlative data suggest that anti-CD19 CAR T cell therapy is well tolerated and highly effective. Moreover, anti-CD19 CAR T cell therapy induces sustained drug-free remission in SLE patients.^8^ To further our understanding of the potential correlates of remission, we performed high-dimensional analysis of SLE patient sera prior to and 3 months following anti-CD19 CAR T cell infusion.

## Results

### Impact of CD19 CAR T cell therapy on circulating serum cytokines in SLE patients

**Figure 1:**
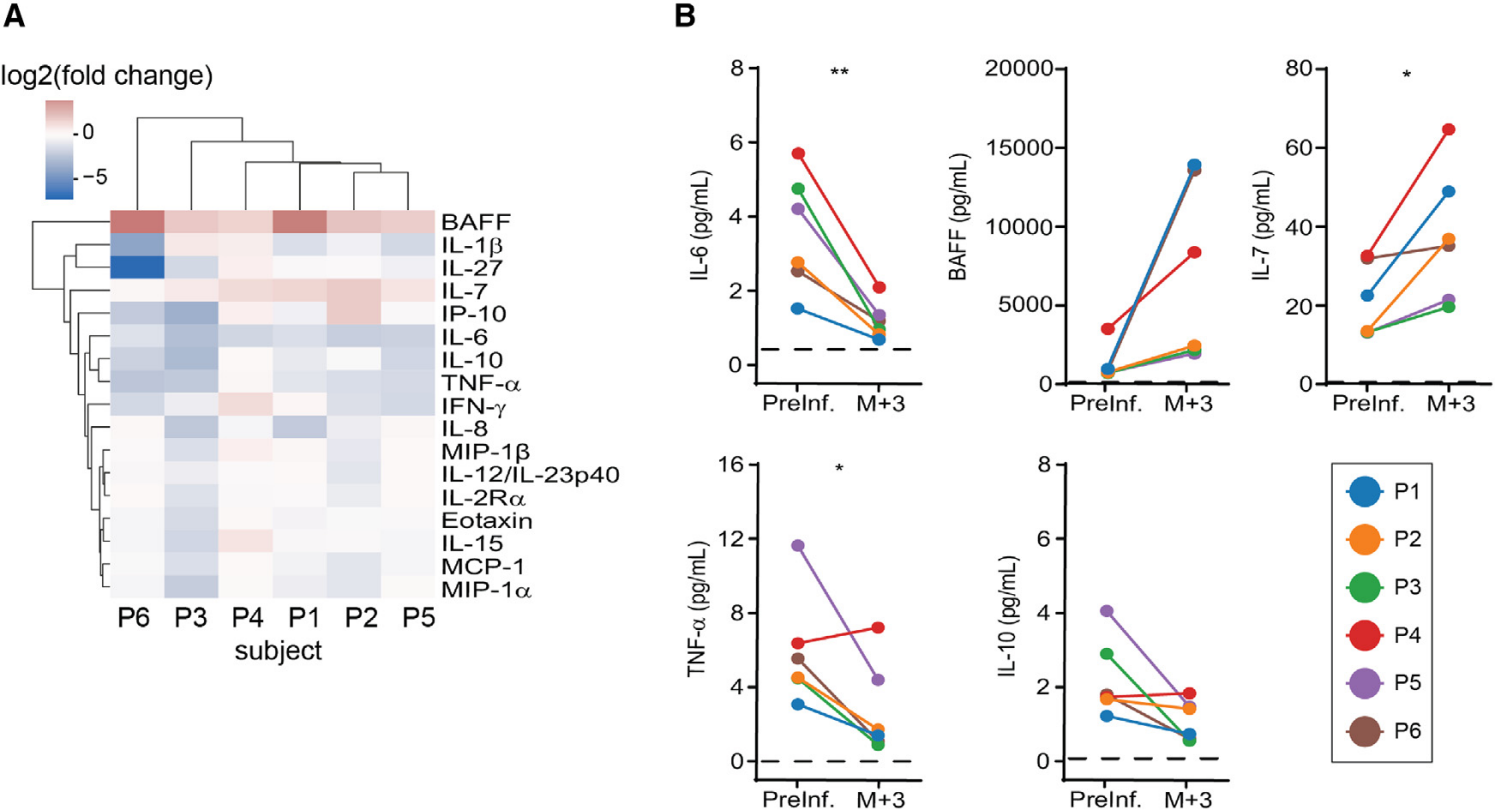
Global changes in systemic cytokines following anti-CD19 CAR T cell therapy in SLE patients (A) Patients and cytokines are ordered based on hierarchical clusters formed using the minimum Euclidian distance between all points in each pair of clusters. Values displayed are the log_2_ transformed ratios of cytokine concentration changes from pre-infusion to the third month after infusion (log_2_ fold change). (B) Concentrations of IL-6, IL-7, IL-10, BAFF, and TNF-α prior to and 3 months following CAR T cell infusion. Dashed black line depicts lower limit of cytokine quantification. P, patient. For pairwise comparisons, ∗p < 0.05, ∗∗p < 0.01, ∗∗∗p < 0.005, Student’s paired t test.

To better understand the role of anti-CD19 CAR T cell therapy on the inflammatory landscape of SLE, we profiled the serum of six patients with refractory SLE treated with CAR T cells for inflammatory cytokines. Sera were collected prior to and 3 months following T cell infusion. The concentration of 25 cytokines in the serum was measured using an electro-chemiluminescence immunoassay (MSD). Interleukin (IL)-1β, IL-2, IL-4, IL-5, IL-12 (IL-12p70), IL-13, IL-21, IL-23, granulocyte-macrophage colony-stimulating factor, and macrophage inflammatory protein-1α were expressed at levels above the limit of quantification for the assay in fewer than nine of the 12 total samples analyzed. For the 15 remaining cytokines, we calculated the log_2_ fold change from baseline to 3 months post-infusion. Unsupervised hierarchical clustering analysis showed that most patients experienced similar changes in serum cytokines following anti-CD19 CART therapy (Figure 1A). Three months after CAR T cell infusion, an increase in the B cell homeostatic cytokines IL-7 (p = 0.0198) and BAFF (p = 0.0518) were observed in all six patients (Figure 1B). The concentrations of IL-6 (p = 0.005), IL-10 (p = 0.036), and tumor necrosis factor (TNF)-α (p = 0.056) were reduced in the sera of all six patients 3 months after infusion (Figure 1B).

### Changes in SLE-associated antibodies following CD19 CAR T cell therapy

**Figure 2:**
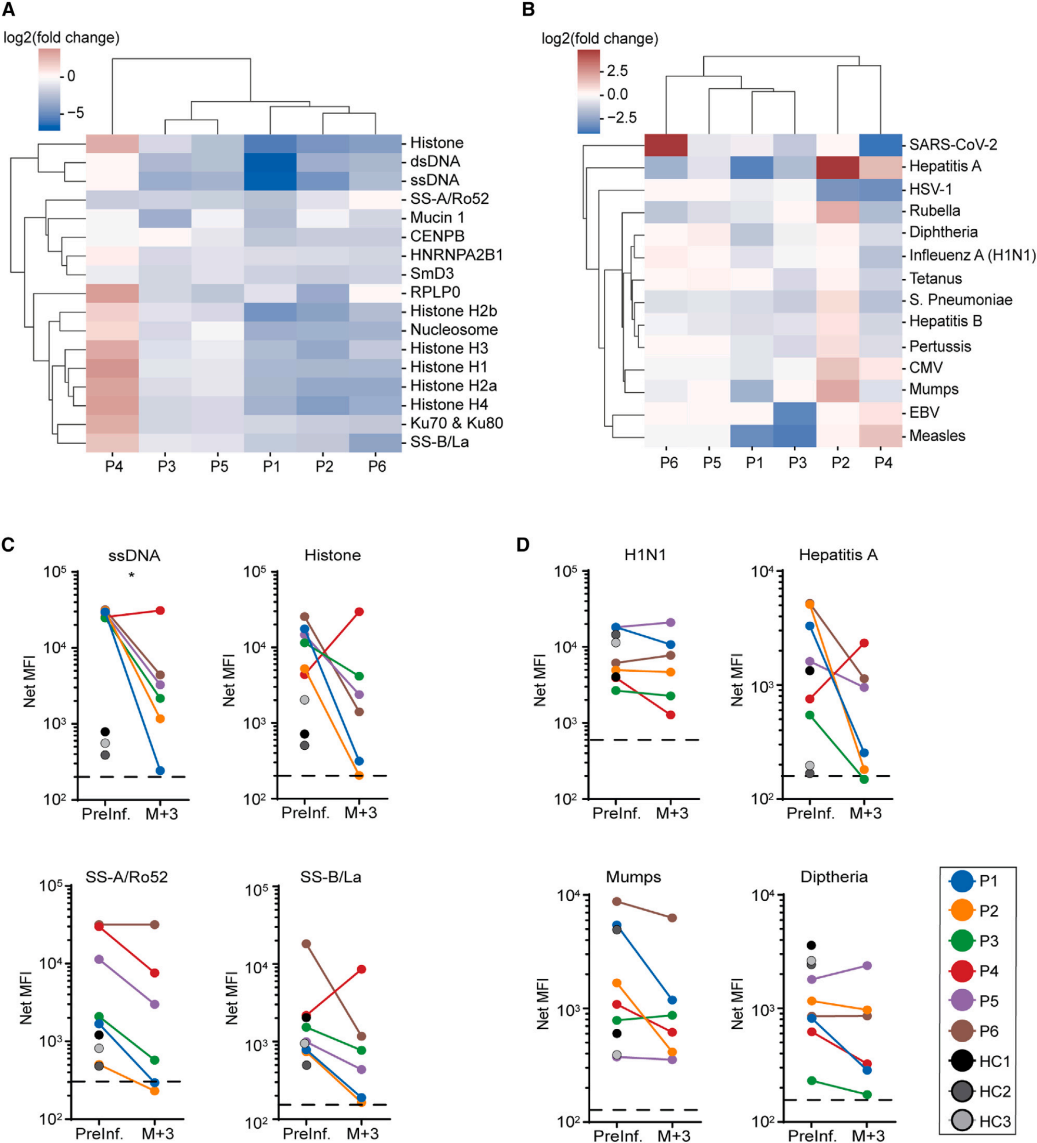
Effects of anti-CD19 CAR T cell therapy on humoral immunity in SLE patients (A) SLE-associated and (B) infectious agents or pathogen-associated antibodies prior to and 3 months following anti-CD19 CAR T cell infusion. Antibodies are ordered based on hierarchical clusters formed using the minimum Euclidian distance between all points in each pair of clusters. Values displayed are the log_2_ transformed ratios of antibody concentration changes from pre-infusion to the third month after infusion (log_2_ fold change). (C) Quantification of antibodies against single-stranded (ss) DNA, total histone, Sjogren’s syndrome (SS)-A/Ro52, and SS-B/La at baseline and 3 months after CAR T cell infusion (N = 6). (D) Quantification of antibodies against H1N1, hepatitis A, mumps, and diphtheria prior to and 3 months following CAR T cell infusion (N = 6). HC, healthy donor control; MFI, mean fluorescence intensity; P, patient. Dashed black line depicts lower limit of antibody quantification.

Patient sera were also evaluated for 17 autoantibodies that have been associated with SLE using a highly sensitive multiplexed immunoassay (Luminex) (Figure 2A).^9^ Anti-DNA antibodies are used to diagnose SLE and correlate to disease severity.^10^ We observed a substantial reduction of anti-double-stranded DNA (dsDNA) and anti-single-stranded DNA (ssDNA) antibodies in five out of six patients 3 months following CAR T cell infusion (Figures 2C and S1). Patient 4 did not have a reduction in anti-dsDNA or anti-ssDNA antibodies (Figures 2C and S1). Reductions in autoantibodies against nucleosome, SSB, HRNPA2B1, Ku70/80, RPLP0, and histone variants (total histone, H1, H2a, H2b, H3, H4) were observed in all patients except for patient 4 (Figures 2C and S1). Anti-SmD3 antibody levels decreased in all patients following cell infusion (Figure S1). Five out of six patients had a moderate reduction in anti-SSA (Ro52) antibodies (Figures 2A and 2C). Only patient 3 had appreciable levels of anti-Mucin and anti-CENPB autoantibodies at baseline, which decreased after infusion (Figure S1).

### Pre-existing humoral immune responses are unaffected by CD19 CAR T cell therapy

Finally, to better understand the impact of CD19^+^ B cell depletion on pre-existing humoral immunity in SLE patients, we assessed patient sera for antibodies to 14 different infectious agents and vaccines. Patient results ranged from no change to mild to moderate decreases in pathogen- or vaccine-associated antibody titers following anti-CD19 CAR T cell infusion (Figure 2B). Importantly, no titers became negative (Figures 2D and S2). An elevation in SARS-CoV-2 antibodies was observed in the sera for patient 6 (Figure S2).

## Discussion

Adoptive transfer of anti-CD19 CAR T cells have demonstrated remarkable efficacy across B cell hematologic malignancies.^11^ Recently, anti-CD19 CAR T cells have been explored in a small number of refractory SLE patients with promising results. As of this report, all six patients reported on herein have achieved and remain in drug-free remission (G. S., unpublished data).^8^^,^^12^ Although these results are impressive, the correlates and mechanisms underlying response are unclear. In this report, we expand on these previous findings by analyzing the changes in circulating serum cytokines and antibodies following anti-CD19 CAR T cell infusion.

We observed increases in circulating serum BAFF and IL-7 in SLE patients 3 months after CAR T cell infusion (Figures 1A and 1B). BAFF is produced by different cell types including monocytes, macrophages, dendritic cells, epithelial cells, and neutrophils.^13^ The TNF surface receptors BAFF-R, TACI, and BCMA bind BAFF and are expressed at various developmental stages within the B cell lineages. BAFF-R and TACI are primarily on CD19^+^ splenic populations, whereas BCMA expression is found both on CD19^+^ B cells and long-lived CD19^−^ plasma cells.^14^ IL-7 can be produced by fetal liver cells, bone marrow stromal cells, thymic stromal cells, and epithelial cells.^15^ Its cognate receptor, IL-7R, is expressed on a variety of different leukocyte populations, including CD19^+^ pro- and pre- B cells, T cells, and innate lymphoid cells.^15^ The observed increase in these cytokines is likely attributable to the complete, but transient, depletion of the CD19^+^ B cell compartment in treated patients. Alternatively, the increase in IL-7 could also be influenced by decreases in either the total number of IL-7R^+^ memory T cells, a decrease in the level of IL-7R expression on memory T cells, or some combination of both in conjunction with the transient loss of CD19^+^IL-7R^+^ pre- and pro- B cells. These shifts in the total number of IL-7R^+^ memory T cells or in IL-7R expression on T cells could also be ascribed to the loss of CD19^+^ antigen-presenting cells. Increased systemic BAFF levels have been observed in SLE patients with active disease.^1^^,^^16^ However, our results show that targeted CD19^+^ B cell depletion is sufficient to further increase serum concentrations of BAFF in this cohort of patients in remission. We predict that systemic levels of BAFF and IL-7 will return to baseline levels as the CD19^+^ B cell compartment is restored after CAR T cells contract.

Interestingly, we also observed decreases in systemic TNF-α, IL-10, and IL-6 (Figures 1A and 1B). Similar to the changes in BAFF and IL-7, these phenomenon could also be attributed to CAR T cell-mediated B cell depletion, as B cells can secrete these factors.^16^ Alternatively, but not mutually exclusive, is that decreased T cell activation resulting from the loss of an important reservoir of professional antigen-presenting cells could manifest in the decreased production of these cytokines. All three of these cytokines are elevated in SLE patients and, along with other interferons, specifically IFNα, drive the interferon signature reported in this patient population.^17^ Their reduction suggests reduced systemic inflammation and aligns with the clinical remission observed in these patients.^8^ Mechanistically, the decreases in TNF-α, IL-10, and IL-6 are aligned with the previously reported drop in serum IFNα in these patients following CAR T cell infusion.^8^

With respect to SLE-associated antibodies, most, but not all, patients exhibited a decrease in circulating SLE antibodies following CAR T cell infusion (Figures 2A and 2C). Interestingly, although all patients were in drug-free remissions at 3 months post-infusion, many of patient 4’s SLE-associated antibodies did not decrease following anti-CD19 CAR T cell infusion (Figures 2A and S1).^8^ Prior assessment of patient 4’s serum via RIA and ELISA were negative for many SLE-associated antibodies, including anti-dsDNA, anti-ssDNA, anti-Sm, anti-SSA/Ro52, anti-SSB/La, and anti-histone antibodies.^8^ The discrepancy between the previous and the current results described herein may be attributed to the increased sensitivity of the assay used to evaluate sera in this report. Furthermore, to date, the role of short-lived and long-lived plasma cells in SLE-associated antibody production is unclear.^18^ One hypothesis for the sustained antibody levels is that the majority of patient 4’s SLE-associated antibodies were produced by long-lived CD19^−^ plasma cells. Furthermore, due to limited post-infusion serum sample availability, the temporal kinetics of any SLE-associated antibody changes remain unclear. That being said, for the other five SLE patients, it appears a majority of self-reactive antibodies are likely produced by short-lived CD19^−^ plasma cells or CD19^+^ antibody secreting cells. As more SLE patients are treated with anti-CD19 CAR T cell therapies, the contribution of short-lived, long-lived, CD19^−^, and CD19^+^ antibody secreting populations to autoantibody production may be further elucidated. Last, as CD38 depletion strategies are starting to be explored in refractory SLE,^19^ plasma cell depletion may be an option for patients who are refractory to anti-CD19 CAR T cell therapy. However, the risk of plasma cell depletion to pre-existing protective humoral immunity should be considered against any potential decrease in autoreactive antibody titers.^19^

To assess the broader impact of anti-CD19 CAR T cell therapy on SLE patients, we also evaluated pre-existing humoral immunity. Consistent with prior reports of CD19-directed CAR T cell therapy in B cell malignancies, there appears to be only a mild to modest impact of anti-CD19 CAR T cells on pre-existing humoral immunity in SLE patients (Figures 2B, 2D, and S2).^7^ These findings are in distinct contrast to SLE-associated antibodies, the majority of which decrease post-CAR T cell infusion (Figures 2A, 2B, and S2). From our limited dataset, it appears that there are two distinct antibody secreting cell populations responsible for the production of SLE-associated antibodies and infectious agent and vaccine-associated antibodies. Moreover, it is possible to exploit this difference therapeutically through anti-CD19 CAR T cell therapy. Interestingly, patient 6 had an increase in circulating anti-SARS-CoV-2 antibodies following T cell infusion. This patient was neither SARS-CoV-2 vaccinated in the 3 months following CAR T cell infusion nor known to have SARS-CoV-2 exposure prior to or within the 3-month post-infusion period. However, this was the only patient of the six profiled in this report to receive intravenous immune globulin (IVIg). We infer that the IVIg donor was SARS-CoV-2 vaccinated and/or COVID-19 convalescent.

Last, as CAR T cell therapy was given in the context of lymphodepletion consisting of fludarabine and cyclophosphamide, attribution of the serologic changes and clinical responses to either the cell therapy and/or the chemotherapy necessitates a nuanced view of leukocyte reconstitution following lymphodepletion and CAR T cell infusion. Three weeks following infusion, CD19^−^ leukocyte populations recovered to pre-infusion levels (G. S., unpublished data).^8^^,^^12^ By comparison, CD19^+^ lymphocyte reconstitution, was, on average 126 ± 51 days following CAR T cell infusion^8^ (G. Schett, personal communication). The post-infusion sera were taken from patients 90 days following CAR T cell infusion. As such, we are inclined to attribute the changes in circulating serum cytokines and autoreactive antibodies to CAR T cell-mediated CD19^+^ B cell aplasia as opposed to the impact of lymphodepletion.

In conclusion, our data provide insights into the change in the cytokine profile of SLE patients undergoing anti-CD19 CAR T cell treatment. We show common principles such as elevation of BAFF and IL-7 as well as decreases of IL-6 but also fine interindividual differences that might be used to define different disease courses after anti-CD19 CAR T cell treatment. Furthermore, autoantibodies seem to decrease and even disappear after anti-CD19 CAR T cell treatment with few exceptions. In contrast, infection and vaccination responses remain remarkably stable during the treatment. Ultimately, longer follow-ups in larger patient cohorts will be needed to confirm the quality of changes in cytokine expression and autoantibody formation in patients with SLE being treated with anti-CD19 CAR T cells.

## Materials and methods

### Patients

Patients were recruited and screened as previously described.^8^ Briefly, all six patients with treatment-refractory SLE, who underwent CAR T cell therapy, were recruited at the Department of Internal Medicine 3 (Rheumatology and Immunology) of the Friedrich Alexander University Erlangen-Nürnberg. Patients screened for this compassionate-use program had to have (1) a diagnosis of SLE according to the EULAR/ACR 2019 criteria; (2) signs of active organ involvement; (3) failure to respond to multiple immunomodulatory therapies, including repeated pulsed glucocorticoids, hydroxychloroquine, belimumab, and MMF; and (4) an understanding of the procedure of CAR T cell therapy. All procedures were performed in accordance with the Good Clinical Practice guidelines of the International Council for Harmonization and covered by license 334_18 B of the institutional review board. Patients provided written informed consent according to CARE guidelines and in compliance with the Declaration of Helsinki principles.

### Cell and vector manufacturing

Cells and vector were manufactured as previously described.^8^ Briefly, the investigational medicinal product MB-CART19.1 consisted of autologous anti-CD19 CAR transduced CD4^+^/CD8^+^-enriched T cells, derived from a leukapheresis product and processed by using the CliniMACS Prodigy device. CD4^+^ and CD8^+^CD3^+^ T cells were enriched from the patients’ peripheral blood apheresis product and a total of 1 × 10^8^ cells were used as the starting cell population. The cells were transduced with a self-inactivating (SIN) lentiviral vector expressing an anti-CD19 41BBz CAR (Miltenyi Biotec). Cells were expanded for 12 days under cleanroom conditions at the GMP-certified laboratory of the Universitätsklinikum Erlangen using the CliniMACS Prodigy system (Miltenyi Biotec).

### Lymphodepletion chemotherapy

Patients received lymphodepleting chemotherapy with fludarabine (25 mg/m^2^/day intravenous [i.v.]) on days −5, −4, and −3 and cyclophosphamide (1,000 mg/m^2^/day i.v.) on day −3 before CAR T cell transfer.

### Serum cytokine assessment

Serum cytokine levels were quantified using Meso Scale Discovery (MSD; Rockville, MD) electro-chemiluminescence multiplex platform at Precision for Medicine. Serum samples were taken from patients prior to or the first day of lymphodepletion chemotherapy, day −5 before CAR T cell infusion, and at 3 months following CAR T cell infusion. Human serum samples were blinded and tested in duplicate using the following panels: VPLEX Pro inflammatory panel, VPLEX Cytokine panel 1, VPLEX Chemokine panel 1, VPLEX Th17 panel, UPLEX IL-2Rα, and UPLEX BAFF. An independent validation of all panels was conducted by Precision for Medicine. Serum samples were tested according to the manufacturer recommended 1:4 dilution. VPLEX and UPLEX assays were performed following the MSD recommended protocol.

### Serum antibody assessment

Serum samples were taken from patients prior to or the first day of lymphodepletion chemotherapy, day −5 before CAR T cell infusion, and at 3 months following CAR T cell infusion. Serum antibody titers were quantified using Luminex 200 technology (Luminex Corporation). A total of 17 proteins were selected to assess SLE-associated antibodies and 14 proteins were selected to assess antibodies to infectious disease pathogens and vaccines. Proteins were purchased from Abcam, AcroBio, Azenta, BPS Bioscience, Creative Biolabs, Creative Biomart, Creative Diagnostics, MyBioSource, Novus Biologicals, Sino Biological, and The Native Antigen Company. Proteins were coupled to magnetic carboxylated beads using the Luminex coupling kit according to the manufacturer’s instructions.

Diluted serum samples were co-incubated with master mix containing desired protein-coupled beads for 1 h at room temperature (RT). After washing with PBS/0.05% Tween 20, the beads were incubated with the biotinylated anti-human secondary antibody (Southern Biotech) for 1 h at RT. After washing, the beads finally incubated with Streptavidin, R-Phycoerythrin Conjugate (SAPE) for 1 h at RT. The beads were washed and analyzed using Luminex 200 instrument. Antibody reactivity values are represented as median fluorescence intensity (MFI).

### Data analysis

Heatmaps were used to visualize the changes in cytokine concentrations or antibody values before and 3 months after infusion. We divided these values by their pre-infusion values for each patient and took the log_2_ transform of the ratios. We then performed hierarchical clustering of the log-transformed ratios using the unweighted pair group method with arithmetic mean (UPGMA) algorithm implemented in seaborn’s clustermap (seaborn.pydata.org) method with Euclidean distance as the distance metric. We used the paired t test implemented in scipy’s ttest_rel method (scipy.org) to calculate p values.

## Data Availability

All data are available in a supplementary Excel file.

## Supplemental information

Document S1. Figures S1 and S2

Table S1. Unprocessed data from serum antibody and serum cytokine evaluation Excel file contains three tabs: SLE, Vaccine, and Cytokine. SLE and Vaccine tabs include antibody quantification listed by antibody and patient. Cytokine tab includes cytokine quantification listed by cytokine and patient. Mean values derived from two technical replicates. MFI, mean florescence intensity; P, patient; HC, healthy donor control for SLE and Vaccine tabs. SLE1, SLE2, SLE3, SLE4, SLE5, and SLE6 refers to P1, P2, P3, P4, P5, and P6, respectively, for Cytokine tab.

Document S2. Article plus supplemental information
