# Supplementary material for: Cytokine and reactivity profiles in SLE patients following anti-CD19 CART therapy
Source: Mol Ther Methods Clin Dev. 2023 Sep 1;31:101104. doi: 10.1016/j.omtm.2023.08.023 (PMC10514439; doi:10.1016/j.omtm.2023.08.023)
Supplement: Document S1. Figures S1 and S2 [file mmc1.pdf]

OMTM, Volume *31*

Supplemental information

Cytokine and reactivity profiles in SLE patients following anti-CD19 CART therapy

Daniel Nunez, Darshil Patel, Jenell Volkov, Steven Wong, Zachary Vorndran, Fabian Müller, Michael Aigner, Simon Völkl, Andreas Mackensen, Georg Schett, and Samik Basu

Figure S1

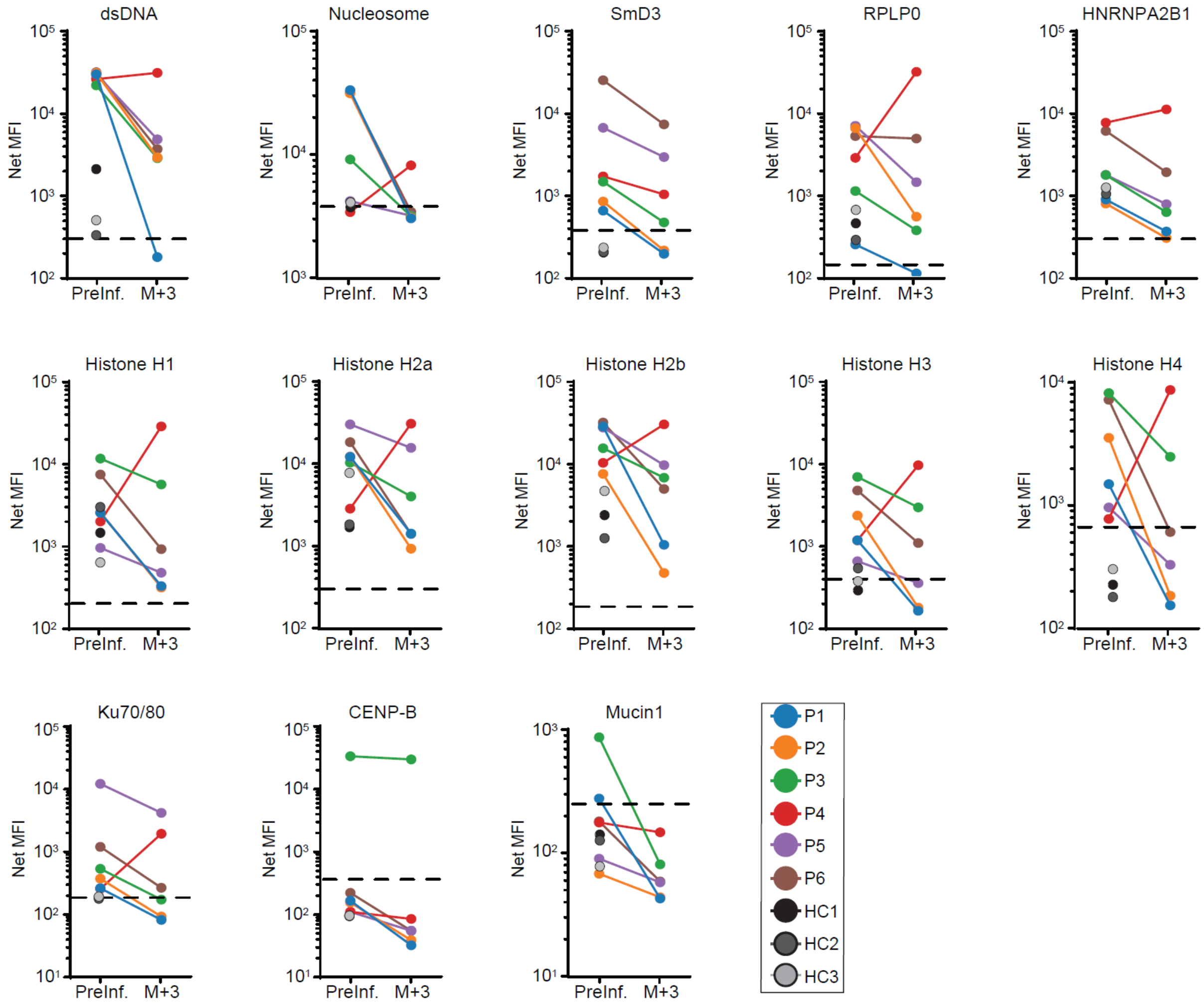

Figure S1. **SLE-associated antibodies before and after anti-CD19 CAR T-cell therapy.** Quantification of antibodies against double stranded (ds) DNA, nucleosomes, Smith (Sm), RPLP0, HNRNPA2B1, Histone variants (H1, H2a, H2b, H3 and H4), Ku70/80, CENBP, and Mucin antigens, prior to and 3 months following CAR T-cell infusion (N = 6). MFI = Mean Fluorescence Intensity. Dashed black line depicts lower limit of antibody quantification. P – patient; HC – healthy donor control

Figure S2

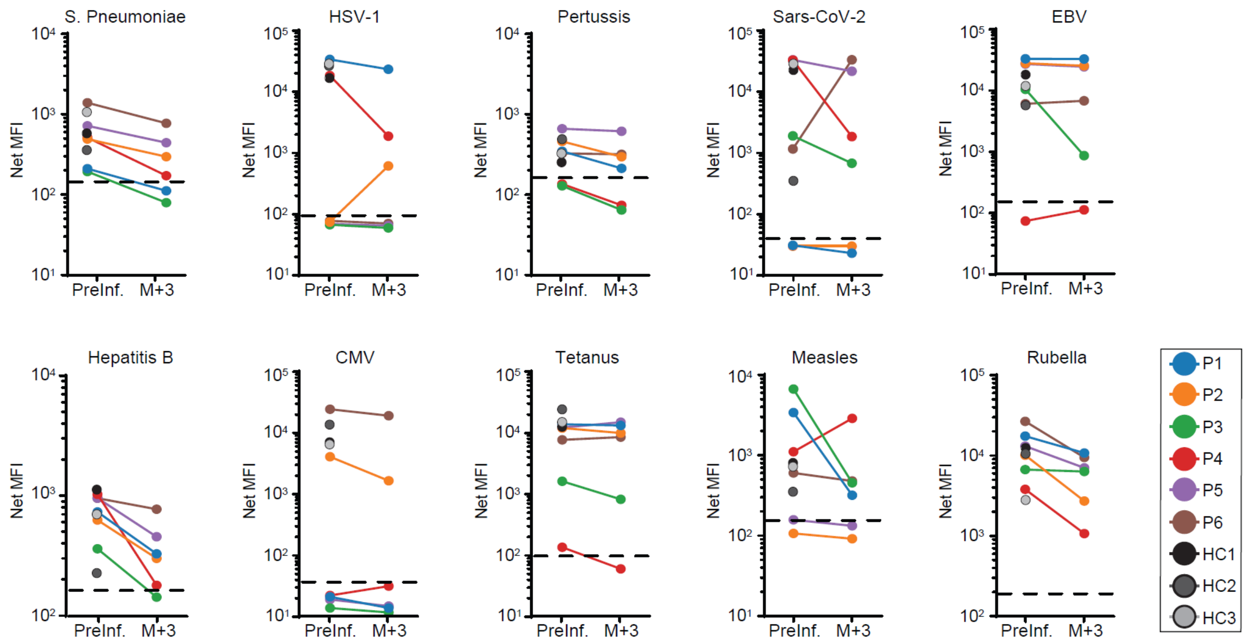

Figure S2. **Infectious agents and vaccine-associated antibodies before and after anti-CD19 CAR T-cell therapy.** Quantification of antibodies against Streptococcus Pneumoniae, Herpes Simplex Virus (HSV-1), Bordetella Pertussis, SARS-CoV-2, Epstein-Barr virus (EBV), Hepatitis B, Cytomegalovirus (CMV), Tetanus, Measles, and Rubella, prior to and 3 months following CAR T-cell infusion (N = 6). MFI = Mean Fluorescence Intensity. Dashed black line depicts lower limit of antibody quantification. P – patient; HC – healthy donor control

Table S1. **Unprocessed data from serum antibody and serum cytokine evaluation.** Excel file contains three tabs: SLE, Vaccine, and Cytokine. SLE and Vaccine tabs include antibody quantification listed by antibody and patient. Cytokine tab includes cytokine quantification listed by cytokine and patient. Mean values derived from two technical replicates. MFI = Mean Florescence Intensity. P – patient; HC – healthy donor control for SLE and Vaccine tabs. SLE1, SLE2, SLE3, SLE4, SLE5, and SLE6 refers to P1, P2, P3, P4, P5, and P6, respectively for Cytokine tab.
